# Characteristics and mental health of psychedelic mushroom and multi-psychedelic users relative to non-psychedelic users in American adults, 2020–2021

**DOI:** 10.3389/fpsyt.2025.1508811

**Published:** 2025-03-05

**Authors:** Sofia Abramsky-Sze, Elliot Marseille, Richard Matzopoulos, Robert Morlock, Leonard Lerer

**Affiliations:** 1 Collaborative for the Economics of Psychedelics, University of California, Berkeley, Berkeley, CA, United States; 2 Division of Public Health Medicine, Faculty of Health Sciences, University of Cape Town, Cape Town, South Africa; 3 Burden of Disease Research Unit, South African Medical Research Council, Cape Town, South Africa; 4 YourCareChoice, Ann Arbor, MI, United States; 5 Back of the Yards Algae Sciences - Parow Entheobiosciences, Chicago, IL, United States

**Keywords:** psychedelics, psilocybin, mental health, anxiety, depression, psychedelic mushroom

## Abstract

**Introduction:**

Few population-based studies have examined associations between psychedelic use and mental health outcomes. This work describes characteristics of exclusive psychedelic mushroom use (referred to as PM use), PMs in combination with other psychedelic substances (multi-psychedelic or MP) use, and non-psychedelic use and explores mental health ratings in non-clinical settings.

**Methods:**

This work uses cross-sectional survey data from American adults collected by Acumen Health Research Institute, including demographic characteristics, general health-related quality of life (Veterans RAND derived mental and physical health composite scores), depression (PHQ 9-item), anxiety (GAD 7-item), comorbid conditions (CCI), health resource utilization, and perceptions, knowledge, and use of psychedelics. Multivariate and descriptive statistics were used to describe participant characteristics. Correlation analysis assessed anxiety and depression scores across groups. Mean anxiety and depression scores were compared using ANOVA and Tukey’s HSD. A multivariate linear regression model controlling for past-year depression, past-year anxiety, age, region, ethnicity, sex, educational attainment, employment, and psychedelic use predicted mental health composite scores (MCS).

**Results:**

Of the 6,869 participants included in the dataset, 256 (3.7%) reported using psychedelics in the last 12 months. Of those using psychedelics, 122 (47.7%) reported PM use and 134 (52.3%) reported MP use. All psychedelic users reported lower MCS and higher levels of anxiety and depression relative to non-users (those who said they had not used psychedelics in the past year). The lowest mental health scores were reported in the MP users followed by the PM users (higher MCS corresponded to better mental health). When controlling for confounding characteristics including past-year anxiety and depression, disparities in mental health scores persisted between those with any psychedelic use and the non-psychedelic group (p<0.001).

**Conclusion:**

This paper extends previous work describing the association between psychedelic use and mental health, controlling for confounding mental health factors such as comorbid anxiety and depression. These results suggest psychedelic users may have poorer mental health than their non-using counterparts in certain contexts and emphasize the need for future research in this field. Both non-adjusted and adjusted analyses demonstrate lower mental health scores for PM and MP users relative to non-psychedelic users. These differential effects highlight the need for further detailed, population-based research on the use of exclusive psilocybin and on psychedelics in combination.

## Introduction

In the United States of America, the last few years have seen a resurgence of interest in psychedelic use, which has manifested in growing numbers of clinical trials as well as widespread media attention. Recent research has shown the potential of psychedelics for a range of neuropsychiatric disorders (1). In the 1950s and 1960s, the field of Western psychedelic research first emerged, with the National Institute of Mental Health sponsoring research into possible medical uses of these drugs. Preliminary results for serotonergic psychedelics (largely Lysergic Acid Diethylamide or LSD) indicated potential benefits for depression and existential suffering among cancer patients (2). However, factors including political backlash to the counterculture movement of the 1960s and pervasive non-medical drug use contributed to the Schedule 1 classification of many psychedelics (which applies to drugs with no currently accepted medical use and a high potential for abuse), hampering research in the field for several decades (3). Along with the Schedule 1 classification, there is also a lack of federal funding in the United States—from 2006-2020, the National Institutes of Health did not directly fund any psychedelic-assisted therapy clinical trials (4).

In recent years, there have been several largely positive results in clinical trials related to psychedelic-assisted psychotherapy. For example, 3,4-methylenedioxymethamphetamine (MDMA)—which can act as both a stimulant and a psychedelic—has been shown to significantly reduce symptoms in patients with severe post-traumatic stress disorder (PTSD) (5). Additionally, in a review of clinical trials, ketamine-assisted psychotherapy was found to have been associated with reductions in anxiety and depressive symptoms (6).

Psilocybin, the main psychoactive component in hundreds of species of psychedelic mushrooms (PMs) has garnered particular scientific interest and positive public perception (7). In clinical settings, PMs have shown overwhelmingly positive effects–in a review of controlled studies, psilocybin use showed potential in treating depression, anxiety, alcohol use disorder, and nicotine addiction (8). Further, in a recent randomized clinical trial, psilocybin-assisted therapy correlated with reductions in depression severity in patients with major depressive disorder (9).

PMs also carry the reputation of being among the safest recreational drugs in terms of low rates of emergency medical treatment (10). However, much of the research into psilocybin use and psychedelic use more broadly has occurred in clinical trial settings (often in conjunction with therapy), and there is a need for more research in naturalistic settings, where key differences can occur. For example, clinical trials use pharmaceutical-grade psilocybin, whereas PMs in naturalistic settings contain compounds beyond psilocybin.

While there is preliminary evidence to support associations between clinical psilocybin use and mental health and well-being (11), mood (12), and anxiety measures (13), there is also limited evidence to suggest that the effects are more varied in naturalistic settings. In a study of individuals with bipolar disorder, one third of respondents reported adverse effects after use of psilocybin (14). Similarly, in a study of psilocybin users who had experienced challenges after consuming PMs, several adverse effects were reported, including putting themselves or others at risk of physical harm. However, the majority of those participants (84%) still believed they had benefited from the experience (15). Much of the aforementioned research into psychedelics focuses on the mental health of psychedelic users over time, rather than comparing them to the general population among health measures.

While cross-population data is limited, some research is beginning to emerge in this field—a recent study found that participants who used hallucinogens for self-described medicinal reasons reported poorer mental health than participants who did not use hallucinogens and those who used hallucinogens exclusively recreationally. Increased depression severity was also linked to an increased likelihood of medicinal hallucinogen use, compared to exclusive recreational use. This finding indicates that reasons for hallucinogen use may be an important correlate for mental health (16).

To better understand factors associated with PMs, Matzopoulos et al. conducted a cross-sectional survey of American adults, collecting demographic data, reasons for psychedelic use, knowledge of psychedelics, various measures of health status, and health utilization (17). The authors found that PM users reported higher rates of anxiety, depression, and healthcare utilization compared to their non-user counterparts, as well as lower current mental health status.

Although the possible association between hallucinogen use and adverse outcomes has been observed as recently as 2024 (16), it is important to note that Matzopoulos’ et al.’s study between November 2020 and March 2021 overlapped with both the height of the COVID-19 pandemic and a time of political turmoil within the United States. In a study using data from a similar time period, Balaet et al. found that those who primarily used psychedelics and cannabis during the pandemic had worse mood-self assessment and resilience scores compared to those who primarily used cannabis or never used drugs (18).

This work expands on this earlier analysis, contributing to the broader literature of psychedelic research as well as the mental health of various groups during the COVID-19 pandemic. At the time of reporting, PM users had lower mental health composite scores (MCS), as well as greater rates of past-year anxiety and depression (assessed via a past-year comorbidity index), compared to the non-psychedelic using group. We hypothesize that reported past-year depression and anxiety is an important confounding variable for disparities in MCS scores between groups, e.g., people with past-year negative mental health experiences may be more likely to a) use PMs and b) have worse mental health at the time of data collection.

We also examine data on multi-psychedelic (MP) users that were not assessed by Matzopoulos et al—this group had taken psilocybin as well as at least one other psychedelic-like substance within the reporting period. We hypothesize that PM and MP users will share more demographic characteristics, mental health results, and views on psychedelics with each other than they will with the non-psychedelics-using group. We hypothesize that MP users may have lower mental health scores relative to PM users, who in turn will have lower mental health scores relative to non-users.

We used multivariate methods to describe the associations between psychedelic use and overall mental health while controlling for the confounding mental health factors of past-year anxiety and depression.

## Methods

### Data collection

Acumen Health Research Institute (AHRI) collected online cross-sectional survey data of adults in the United States (US). They utilized a random stratified sampling framework to ensure a sample with a demographic composition similar to that of the US adult population by region, gender, age, and race. The survey was Institutional Review Board-exempt, as responses were both anonymized and aggregated. Between November 2020 and March 2021, participants were recruited monthly through AHRI’s monthly online research panels. The AHRI survey was sent to 8,500 participants with 7,139 individuals completing the assessment and included in the dataset (response rate of 83.8%). Further information on the survey methodology for this study is provided in Matzopoulos et al, and in similar AHRI studies (19–21).

### Psychedelic use survey questions

The initial survey included three questions for all participants regarding psychedelics. The following categories were classified as psychedelics in the initial survey: PM, ayahuasca, DMT, 5-MEODMT, ibogaine, kambo, ketamine, LSD, MDMA, and peyote. Participants were first asked about their knowledge of the potential benefits of psychedelics: *“Have you heard of psychedelic use for any of the following? Select all that apply.*” Response options included: general mental health and well-being when feeling basically satisfied with life or for personal development; managing a diagnosed psychiatric condition (depression, PTSD addition, etc.); to address a specific worry/concern in your life (e.g., relationship issue, bereavement, addiction, trauma); no knowledge, and other (please specify). Participants were then prompted with the statement: “*In the past 6 months I have heard more than usual about the positive uses of psychedelic drugs (e.g., magic mushrooms) for mental health issues (depression, PTSD, addition, etc.)*.” This statement was a 5-point Likert Scale with response options from “strongly disagree” to “strongly agree”.

The survey included a question about psychedelic use over the past 12 months: “In the last 12 months, have you used any of the following? Select all that apply.” Participants could choose “None of these” or select one or more options from the list of psychedelics. For each psychedelic selected, participants were asked follow-up questions about their experiences in the past year. The first follow-up question was: “*In the last 12 months, did you use with the specific intention of improving your: Select all that apply*,” with response options matching those provided in the initial question (e.g., general mental health and well-being). Additional follow-up questions included: *“In the last 12 months, did your use of <the psychedelic> increase, decrease, remain unaffected, or other (please specify)” due to COVID-19 or election-related factors.* Finally, participants were asked whether they sought emergency medical treatment following their use of the psychedelic (yes/no response options).

### Mental health variables

Participants self-reported demographic characteristics, health status, educational attainment, employment, and past-year use of psychedelics. The Veterans RAND-12 assessment, a widely used patient-reported outcome instrument, was used to assess current composite mental health (MCS), physical health, and overall health utility (22). Recent anxiety was assessed with the Generalized Anxiety Disorder 7-Item Scale (GAD-7) while depression was assessed using the Patient Health Questionnaire 9-Item scale (PHQ-9). Comorbidities, including past-year anxiety and depression, were measured through the Charlson Comorbidity Index (CCI), which was calculated from self-reported conditions. The CCI is used to predict mortality for patients with a range of comorbid conditions and has been shown to be both a highly valid and highly reliable instrument (23). The codebook with all questions provided to participants can be found in the supplemental section.

### Analysis

A new variable, “anxiety/depression” was created to combine scores from the anxiety and depression variables of the CCI. Participants with past-year anxiety and/or depression were compared to participants without either condition across descriptive characteristics, including age, sex, race, educational attainment, region, employment status, health insurance, MP use, and PM use. Chi-Square analyses were used to compare categorical variables and ANOVA for continuous variables.

Data were further stratified into 3 groups based on past-year psychedelic use: participants who had not used psychedelics, participants (PM group) whose psychedelic use was confined to psychedelic mushrooms, and participants (MP group) who had used PM and other psychedelic substances. The remaining individuals, participants with non-PM psychedelic use, were excluded from data analysis. ANOVAs were carried out to assess MCS by psychedelic use and past-year anxiety or depression. These ANOVAS were followed by post-hoc Tukey’s HSD tests and Bonferroni’s correction. A multivariate linear regression model controlling for past-year depression, past-year anxiety, age, region, ethnicity, sex, educational attainment, employment, and psychedelic was used to assess factors related to MCS.

## Results

7,139 individuals were included in the original dataset. Of these, 6,869 were included in our analyses (270 responses were excluded for past-year use of other psychedelics with no past-year use of PM). We compared the 4,016 adults without past-year anxiety or depression to the 2,853 adults with past-year anxiety or depression (**Table 1**).

**Table 1:** Comparison of individuals with past year anxiety or depression to individuals without past-year anxiety or depression.

	Overall survey population	Without anxiety or depression in last 12 Months	With anxiety and/or depression in last 12 Months	p-val
n	6869	4016	2853	
Age [years] (SD)	46.56 (16.75)	50.57 (17.11)	40.92 (14.45)	<0.001
Demographic Factors				
Sex, % male (n)	44.6% (3065)	50.5% (2027)	36.4% (1038)	<0.001
Black, % (n)	15.4% (1061)	17.6% (706)	12.4% (355)	<0.001
White, % (n)	73.9% (5075)	72.1% (2894)	76.5% (2181)	<0.001
Other, % (n)	10.7% (733)	10.4% (416)	11.1% (317)	0.34
Education greater than high school, % (n)	72.3% (4966)	75.5% (3032)	67.7% (1934)	<0.001
Employed Full time, % (n)	30.3% (2079)	33.1% (1329)	26.3% (750)	<0.001
Health Insurance, % (n)	84.8% (5825)	86.6% (3476)	82.3% (2349)	<0.001
Region, % (n)				0.03
Northeast	17.6% (1212)	18.2% (731)	16.9% (481)	
Midwest	22.6% (1553)	22.6% (908)	22.5% (642)	
South	39.5% (2715)	38.2% (1533)	41.4% (1182)	
West	20.3% (1392)	21.0% (844)	19.2% (548)	
Psychedelic Use				
Reporting PM, % (n)	1.8% (122)	1.0% (41)	2.8% (81)	<0.001
Reporting MP, % (n)	2.0% (134)	1.3% (53)	2.8% (81)	<0.001

### MP and PM cohorts

MP and PM users demonstrated significantly higher knowledge of potential benefits of psychedelic use than the non-use group (p<0.001 using z-tests across all groups; p<0.001 for pairwise comparisons between MP and non-use groups and PM and non-use groups). MP users displayed slightly higher knowledge than their PM counterparts across the knowledge variables, but these results were not statistically significant (**Table 2**). For PM users, general mental health and well-being was the most common reason for use, followed by intentions of improving diagnosed psychiatric conditions and intentions of addressing specific worries. For the question regarding an increase in the last six months in hearing about positive uses of psychedelic drugs for mental health issues, the mean MP score was 1.81, compared to 2.06 for PM users and 3.55 for non-users. Since 1 on this scale corresponded to “strongly agree,” and 5 corresponded to “strongly disagree,” these results could indicate increased recent discussion around/knowledge of possible psychedelic benefits among respondents who had partaken in psychedelics.

**Table 2:** Knowledge of potential benefits of psychedelic use among all groups and reasons for PM use among the PM group.

N	Psychedelic use			
	None	PM	Multi	p-val
	6613	122	134	
Has heard of psychedelic use for at least one of the following, % (n)	28.3 (1873)	83.6 (102)	90.3 (121)	<0.001
Psychedelic use for general mental health/well-being when feeling basically OK with life, or personal development, % (n)	16.4 (1082)	63.9 (78)	67.1 (90)	<0.001
Psychedelic use for managing a diagnosed psychiatric condition, % (n)	16.8 (1108)	51.6 (63)	57.4 (77)	<0.001
Psychedelic use to address a specific worry / concern in your life, % (n)	8.0 (530)	35.2 (43)	41.8 (56)	<0.001
Had used PMs for at least one of the following reasons in the past 12 months, % (n)		86.1 (105)		
Improving general mental health / well-being when feeling basically OK with life or for personal development (mean)	–	62.3 (76)	–	
Improving management of a diagnosed psychiatric condition (mean)	–	33.6 (41)	–	
Improving a specific worry / concern (mean)	–	21.3 (26)	–	

### Mental health measures among the three groups

In the overall sample, past-year anxiety and depression were negatively correlated with present mental health status. Furthermore, past-year anxiety and depression were statistically significantly greater among the PM (*OR=2.88; 95% CI [1.98, 4.24])* and MP groups (*OR=2.23; 95% CI [1.57, 3.18]*) compared to their non-use counterparts. 66.39% of PM users had past-year experiences of anxiety and/or depression, compared to 60.45% of MP users and 40.69% of non-psychedelic participants.

**Figure 1:**
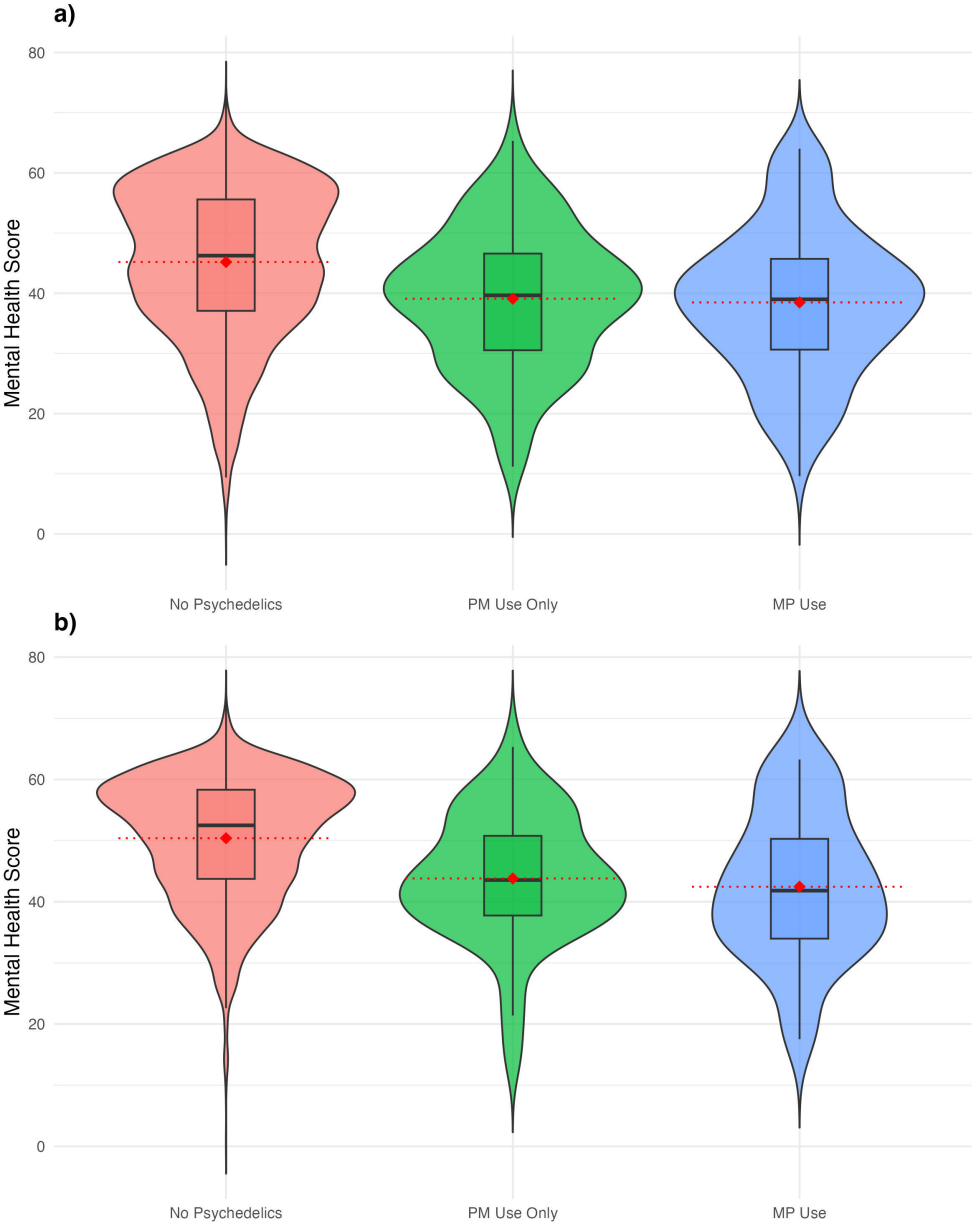
Comparisons of overall Mental Health Composite Scores. **(a)** Comparisons of overall Mental Health Composite Scores for PM users, MP users, and non-users of psychedelics. **(b)** Comparisons of overall Mental Health Composite Scores for PM users, MP users, and non-users of psychedelics without past-year experiences of depression or anxiety. Violin plots showing the mental health composite scores. Violin plots showing the mental health composite scores, stratified by past-year psychedelic use. **(a)** Represents all participants included in the data analysis and **(b)** represents participants without past-year anxiety or depression.

MCS12 scores were lower for those reporting PM use (median 38.40, SD 12.08) and those reporting MP use (median 39.65, SD 11.34) compared to the median of 46.25 and SD of 12.52 for the non-psychedelics group (**Figure 1**). The interquartile ranges for the PM and MP users also reflected each other, with the 75^th^ percentile for those groups mirroring the 50^th^ percentile for the non-psychedelic groups. The total range of scores was the greatest for the non-psychedelic group, which also recorded the largest number of outliers.

The mean MCS for all groups were similar to the median scores, indicating normality (means 38.48 MP, 39.09 PM group, 45.19 non-psychedelic group, p< 0.001). For users without past-year depression or anxiety comorbidities, the means were 42.46 for the MP group, 43.81 for the PM group, and 50.39 for the non-psychedelic group (**Figure 1**). MP and PM MCS were similar to one-another but lower than the non-psychedelic group scores. The violin plot illustrates the comparisons between the three groups; the boxplot illustrates the interquartile range, and the dotted red line illustrates the mean for each of the three groups.

**Figure 2:**
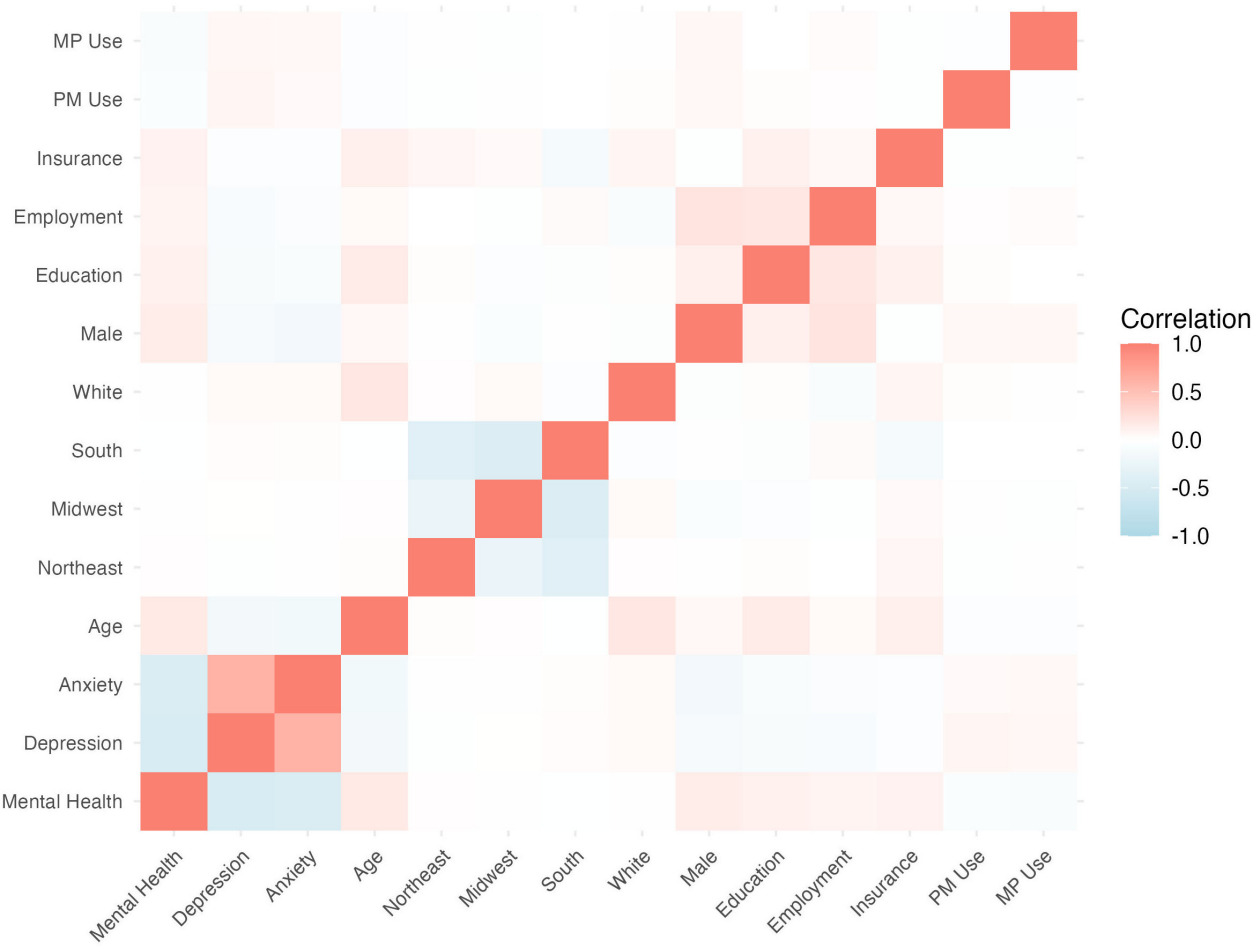
Heatmap assessing correlation between variables in the linear model. Correlation heatmap showing the relationships between variables. The color scale represents the strength of the correlation, with blue indicating a negative correlation, white indicating no correlation, and red indicating a positive correlation. Values closer to -1 or 1 indicate stronger correlations, while values near 0 suggest weaker or no correlation.

The ANOVA analyses showed statistically significant differences in the mean MCS between the three groups. A one-way ANOVA revealed a significant effect of psychedelic use on MCS, F(2,6086)=31.62,p<.001. In the Tukey’s HSD post-hoc analysis, the mean difference for No psychedelics vs. MP use was 6.71 (95% CI [4.16, 9.26], p<.001), indicating a significant difference between these groups. For No psychedelics vs. PM use, the mean difference was 6.09 (95% CI [3.42, 8.77], p<.001), also indicating a significant difference between these groups. Finally, when comparing the PM vs MP groups in the Tukey’s HSD analysis, the mean difference was 0.62 (95% CI [-3.05, 4.28], p=.918), which was not statistically significant. For both the ANOVA and the Tukey’s analysis, the results were consistent when adding anxiety/depression as a blocking variable to address potential confounding (**Table 3**). Using Bonferroni’s correction, there were statistically significant differences in MCS between both PM and MP groups for the total dataset population and for those without past year anxiety and/or depression (p<0.001). However, there were not statistically significant differences among groups for the population with past year anxiety and/or depression. A heatmap illustrated correlations between various demographic and mental health variables (**Figure 2**).

**Table 3:** Test for statistical significance in MCS by group.

ANOVA analysis for MCS by past-year psychedelic use					
	Degrees of Freedom	Sum of Squares	Mean of the Sum of Squares	F Value	Pr (>F)
Psychedelic Use	2	10169.10	5084.55	32.6	<0.001
Residuals	6866	1070907.35	155.97		

Tukey’s HSD analysis for MCS by past-year psychedelic use					
	Difference	Lower Bound of 95% Confidence Interval	Upper Bound of 95% Confidence Interval	P-value	
No Psychedelics—MP Use	6.71	4.16	9.26	<0.001	
No Psychedelics—PM Use	6.09	3.42	8.77	<0.001	
PM Use—MP Use	0.62	-3.05	4.28	0.92	

ANOVA analysis for MCS by past-year psychedelic use, with anxiety/depression as a blocking variable					
	Degrees of Freedom	Sum of Squares	Mean of the Sum of Squares	F Value	Pr (>F)
Psychedelic Use	2	10169.10	5084.55	45.38	<0.001
Anxiety/Depression	2	301833.7	150916.85	1346.94	<0.001
Residuals	6864	769073.70	112.04		

Tukey’s HSD analysis for MCS by past-year psychedelic use, with anxiety/depression as a blocking variable					
	Difference	Lower Bound of 95% Confidence Interval	Upper Bound of 95% Confidence Interval	P-value	
No Psychedelics—MP Use	6.71	4.54	8.88	<0.001	
No Psychedelics—PM Use	6.09	3.83	8.36	<0.001	
PM Use—MP Use	0.62	-2.49	3.72	0.89	

In the linear model controlling for past-year depression (**Table 4**), past-year anxiety, age, region, ethnicity, sex, educational attainment, employment, and psychedelic use, the following variables were predictive of MCS12 score with p<0.001: past-year depression; past-year anxiety; age; sex; education status, health insurance status; MP use. PM use was also associcated with MCS (p<0.01).

**Table 4:** ANOVA on Linear model assessing MCS.

Predictor	Beta	Standard Error	F.value	P.value
(Intercept)	44.31	0.57	NA	NA
Depression	-8.68	0.35	2426.63	<0.001
Anxiety	-6.28	0.34	414.5	<0.001
Age greater than or equal to 30	2.05	0.36	58.5	<0.001
Northeast	0.5	0.41	0	0.9767
Midwest	0.82	0.39	0.6	0.4377
South	0.82	0.35	1.82	0.1778
White	-0.12	0.3	0.27	0.6028
Male	1.64	0.27	39.15	<0.001
Education greater than high school	0.95	0.29	16.55	<0.001
Employed Fulltime	0.48	0.29	3.27	0.0708
Health Insurance	2.19	0.36	38.61	<0.001
PM Use	-2.81	0.96	7.79	0.0053
MP Use	-3.6	0.92	15.32	<0.001

Among PM users, a weak positive correlation was observed between past-year anxiety and current anxiety scores (r=0.155, p=0.09), and a similar weak but statistically significant positive correlation was found between past-year depression and current depression scores (r=0.227, p=0.01). While the correlation for depression among MP users aligned with that of PM users (r=0.228, p=0.01), the anxiety correlation for MP users were both less weak and statistically significant (r=0.228, p=0.01). Finally, non-users exhibited moderate correlations between both anxiety (r=0.481, p<0.001) and depression (r=0.495, p<0.001) variables.

## Discussion

The objective of this paper was to expand on the prior research conducted by Matzopoulos et al., which found a potential association between PM use and negative health outcomes, by exploring possible confounding variables and by adding the analysis of outcomes in MP users—those who had used PMs in addition to at least one of the other psychedelic-like substances listed in the methods section. This survey found that men were more likely than women to use psychedelics, which is in accord with previous findings that men are more likely than women to use almost all illicit substances (24). Additionally, the age differences between groups are unsurprising, as younger generations typically possess more accepting attitudes towards psychedelics (25).

In this dataset, the total prevalence of past-year psychedelic use was 7.4%, equivalent to 19 million American adults (7.4% includes the participants who were excluded from the analysis for past-year use of psychedelics without past-year use of PMs) (26). However, the 2021 National Survey on Drug Use and Health (NSDUH) reported less than 3% of individuals aged 12 or older had engaged in past-year hallucinogen use (27) The higher rates of reported psychedelic use could be because individuals who had utilized substances might be more inclined to a) respond to a survey on psychedelics and b) answer specific questions pertaining to psychedelic use. Surveys such as the NSDUH, which sometimes utilize in-person reports, may be subject to underreporting due to respondents’ fear of identification or stigmatization due to drug use (28).

PM users and MP users reported higher levels of anxiety and depression compared to the non-psychedelic group. Additionally, the MP group consistently reported lower mental health scores than the PM group, although both had worse mental health compared to the non-users. The observed higher levels of anxiety and depression among both PM and MP users compared to non-psychedelic user participants underscore the importance of understanding the complex relationship between psychedelic use and mental health outcomes, especially in complex social or political contexts.

Our findings on the reasons for psychedelic use highlight that the average PM user had reported utilizing PM for at least one of the following reasons: general mental health/well-being, addressing diagnosed psychiatric conditions, or managing more specific worries. This highlights the potential therapeutic and personal development reasons behind psilocybin use.

The motivations behind PM use could align with prior reports of psilocybin having a relatively low toxicity profile and high potential for clinical use (29). Psychedelic science—specifically in the context of psilocybin-assisted therapy—offers a promising avenue for innovative treatments for a variety of conditions. There is a large body evidence that clinical and guided use of psilocybin, in conjunction with existing treatments, can lead to improved patient outcomes, although the exact duration of these positive effects is still largely unknown. In clinical settings, psilocybin has been associated with reductions in depressive symptoms (30, 31), abstinence in alcohol-dependent participants (32), and long-term smoking cessation (33). Interestingly, one of the above studies noted adverse physical effects such as headache and nausea associated with psychedelic use, while the other authors did not mention similar findings.

While the clinical landscape is increasingly demonstrating positive results, most use takes place outside the clinical setting. Research conducted in naturalistic settings has also found evidence to support associations between psilocybin use and mental health metrics; these include well-being (11), mood (34), and anxiety measures (13). The positive evidence found in these studies is encouraging for those involved in the field.

Fascinatingly, there are studies where adverse effects of psilocybin have been reported, but participants still endorsed their experiences. In a study of individuals with bipolar disorder, the adverse effects following psilocybin use included manic symptoms, difficulty sleeping, and anxiety (14). Similarly, in a study of 1993 psilocybin users who had experienced a challenging experience after consuming PMs, several adverse effects were reported, some of which were not limited to an acute timeframe. Of those whose experience had occurred over 1 year prior to data collection, 7.6% had sought treatment for enduring psychological symptoms, indicating potential long-term negative effects (15). However, in both of these studies, the participants on the whole believed they had benefited from the experience or that it was more helpful than it was harmful. These reports indicate that the effects of PM use are incredibly complex —they may be associated with adverse effects and still subjectively rated as a beneficial experience.

Interestingly, psilocybin in combination with other substances has been linked to occurrences of long-term negative health outcomes (35). While not specific to psilocybin, hallucinogen-associated hospitalizations have shown a large relative but small absolute increase since 2016 in California (36). Overall, nuances continue to emerge within the body of literature on naturalistic psilocybin use, especially in combination with other substances. Future research outside the clinical setting continues to be critical to inform a more holistic understanding of the factors driving psilocybin use, as well as the acute and chronic impacts of said use.

In their initial survey, Matzopoulos et al. found that PM users consistently expressed both worse current mental health and reported higher levels of past-year anxiety and depression. The current paper examines the hypothesis that those with recent anxiety and depression would be both more likely to use PM and more likely to report current worse mental health. When controlling for past-year anxiety and depression, the differences in mental health statuses between groups were still statistically significant (p<0.001). These results hold for multiple comparisons for the total dataset population and for those without past year anxiety and/or depression (p<0.001), but not for the population with past year anxiety and/or depression. This research supports and extends the original findings, suggesting a potential association between non-clinical psilocybin use and poorer mental health among American adults, even when controlling for past mental health experiences.

As Matzopoulos et al. note, this relationship is contrary to findings from an international study, which concluded that regular users of psychedelics had less psychological stress than occasional users and non-users. The authors of that study concluded that psychedelics could either serve as a protective factor or that people with certain traits would be more likely to use psychedelics (37).

The differences in findings could be ascribed to fundamental distinctions between study populations and sampling frames (American users and international counterparts, PM users and psychedelic users at large, any use and regular use). Additionally, much of the research on psilocybin use assesses participants’ mental health before and after psychedelic experiences, an approach which facilitates tracking individual changes in well-being. In other words, psychedelic users may have poor mental health relative to the general population, but psychedelic use is associated with improvements in their baseline mental health. Conversely, because of the cross-sectional nature of our data, it is impossible to determine whether poor mental health preceded PM or MP use. Our study suggests an association between psilocybin use and worse mental health, perhaps due in part to the fact that we compare PM users to the general population, and not to their own baseline state. As mentioned previously, over 60% of the PM users had used PMs with the intent of reporting general mental health or well-being. This finding may suggest the chronology is as follows: PM users have worse pre-existing mental health than the general population, wish to improve their mental health, and use PMs. Subsequently, Even if the PM use temporarily assists with mental health, the metric is lower than that of the non-using population.

The findings from this study have some alignment to the results of Balaet et. al, which found worse mood self-assessment and resilience scores among those who had used psychedelics and cannabis during the COVID-19 pandemic, compared to those who either primarily used cannabis or did not use drugs (18). That study used data from the Great British Intelligence Test, whereas our survey used data from an American population. In combination, these studies may provide evidence as to the mental state of psychedelic users in times of crisis or disruptions to daily life. That is, those who are more likely to use psychedelics may also be especially vulnerable to changes in mental health driven by external contexts such as the pandemic. Although the COVID-19 pandemic was unprecedented, these results could provide grounds for further research into psychedelics and mental health in other extraordinary social or political periods. It is possible that disruptions to daily life may serve as a particular risk factor for the mental health of psychedelic users.

Additionally, our research, as hypothesized, demonstrated a plethora of similarities between MP and PM users. Specifically, the mental health status of those groups was similar, both in terms of MCS and in terms of past-year anxiety and depression. These results address one of the limitations of Matzopoulos et al.’s study, which was that MP users were not included in the analyses. The authors noted that omitting seasoned psychedelic users—those who used more than just psilocybin—might lead to inflated negative mental health reports by PM users (more novice than the MP group and perhaps more susceptible to the effects of psilocybin). However, our findings demonstrate that that is not the case, and PM and MP users both exhibit worse mental health than the non-psychedelic group.

### Limitations

The first limitation of our analyses is that we did not weight the data, although we did control for several different factors. Since the initial authors conducted a cross-sectional survey, it is difficult to assess whether poor mental health preceded PM use or vice versa. Other standard survey limitations apply, especially the electronic format, which ensured the researchers only included participants with enough computer literacy to participate in an online survey. The anonymized format may also have resulted in a self-selecting group of respondents, hence the notably higher rates of psychedelic use than have been found in other reports.

Furthermore, the PM and MP groups each had under 150 participants. Another possible limitation is that the survey terminology included psychedelic mushrooms as a whole instead of referring to psilocybin—Amanita Muscaria mushrooms are also considered to have psychedelic properties, although the experience may differ from the effects associated with psilocybin mushrooms (38). It is possible that a small number of respondents may have been referring to Amanita Muscaria use. Because the initial paper was focused specifically on PMs, the original work did not distinguish between other types of psychedelics when converting the survey responses into a dataset. Therefore, the data available for our follow-up analysis was limited by the classification of the original work. It is important to note that the psychedelic substances included in the paper can have differing pharmacological profiles and may be associated with differential mental health outcomes. The results of this paper may thus not adequately explore the nuances associated with different psychedelic-like substances beyond psilocybin, and may not be as generalizable to broader contexts. Another limitation of the survey data is that the study focused on “any” PM use, rather than “regular” or “frequent” use, which ignores the possible dose-response relationship between PMs and mental health.

Perhaps the largest limitation of the survey was that no data was collected on use of alcohol, nicotine, marijuana, or other non-psychedelic drugs. Given that there may be significant associations between psychedelic use and use of other substances, the lack of this data serves as an important limitation in understanding the effect of this data. In particular, psilocybin use has been found to be associated with past-year Cannabis Use Disorder (39). Non-psychedelic substances could serve as potential confounders and explain some of the findings, and not having that information in the survey inhibits the conclusions we are able to draw from the data.

We also note the political and social context of the survey collection, which occurred between November 2020 and March 2021, at the height of the COVID-19 pandemic. The pandemic resulted in increased rates of anxiety and depression for almost all groups, which may have appeared in the responses seen in the study (40). Additionally, the timing coincided with the 2020 election and January 2021 insurrection, a high-stress political climate which also corresponded with increased levels of anxiety and depression for American adults (41). The unique overlap of these two events may make the survey collection results less generalizable to other time periods. It is possible that current reported mental health status for all groups was lower than it would have been had the data been collected at another time, and past-year anxiety and depression were higher. Similarly, it is possible that these events may have affected the likelihood of substance use among certain groups more than others—this could hold true for both psychedelics (as mentioned in this survey), and for other substances for which survey data was not collected.

### Conclusions

The present research demonstrates the need for further population-based studies on psychedelic use, specifically regarding psilocybin. Although PMs have a positive public perception and have been shown to have strong associations with health benefits in clinical settings, the results of this analysis support and strengthen the findings of Matzopoulos et al., in particular that those who use psychedelics may have poorer mental health than their non-using counterparts. Future psychedelic research should include population-level surveys, investigate the processes observed in the original study, differentiate between psychedelic substances with different pharmacological profiles, and control for non-psychedelic substances in order to better address potential confounders. Research beyond the clinical setting continues to be paramount in understanding the long-term effects of psilocybin (both by itself and in combination with other substances).

## Data Availability

The original contributions presented in the study are included in the article/supplementary material. Further inquiries can be directed to the corresponding authors.
