# Correction: Characteristics and mental health of psychedelic mushroom and multi-psychedelic users relative to non-psychedelic users in American adults, 2020–2021

**DOI:** 10.3389/fpsyt.2026.1834094

**Published:** 2026-04-20

**Authors:** Sofia Abramsky-Sze, Elliot Marseille, Richard Matzopoulos, Robert Morlock, Leonard Lerer

**Affiliations:** 1 Collaborative for the Economics of Psychedelics, University of California, Berkeley, Berkeley, CA, United States; 2 Division of Public Health Medicine, Faculty of Health Sciences, University of Cape Town, Cape Town, South Africa; 3 Burden of Disease Research Unit, South African Medical Research Council, Cape Town, South Africa; 4 YourCareChoice, Ann Arbor, MI, United States; 5 Back of the Yards Algae Sciences - Parow Entheobiosciences, Chicago, IL, United States

**Keywords:** psychedelics, psilocybin, mental health, anxiety, depression, psychedelic mushroom

In the published article, there was an error in the **Generative AI statement**. The statement was incorrectly written as “The author(s) declare that no Generative AI was used in the creation of this manuscript.” The corrected statement is:

"Generative AI (ChatGPT) was used in the preparation of the original manuscript solely to assist with reformatting bibliographic references to Frontiers style. This use was limited to citation formatting, specifically the DOI fields, and did not involve content generation, analysis, or manuscript drafting. Errors introduced in DOI fields during this process have been corrected.”

There were several errors in the **References**:

The DOI for Reference 4 was erroneously written as “doi: 10.1016/j.drugpo.2021.103457”. It should be “doi: 10.1016/j.drugpo.2021.103473”.
The DOI for Reference 6 was erroneously written as “doi: 10.2147/JPR.S371736”. It should be “doi: 10.2147/JPR.S360733”.
The DOI for Reference 8 was erroneously written as “doi: 10.7759/cureus.29953”. It should be “doi: 10.7759/cureus.30214”.
The DOI for Reference 14 was erroneously written as “doi: 10.1177/02698811221132017”. It should be “doi: 10.1177/02698811221131997”.
The DOI for Reference 22 was erroneously written as “doi: 10.1097/JAC.0000000000000416”. It should be “doi: 10.1097/JAC.0000000000000420”.
The DOI for Reference 23 was erroneously written as “doi: 10.1159/000522541”. It should be “doi: 10.1159/000521288”.
The DOI for Reference 25 was missing. “Available online at: https://www.jstor.org/stable/48615348 (Accessed October 01, 2024)” in the original article has been replaced by “doi: 10.1177/1403494817724981”.
The DOI for Reference 28 was erroneously written as “doi: 10.1016/j.annepidem.2022.01.010”. It should be “doi: 10.1016/j.annepidem.2021.12.013”.
The DOI for Reference 34 was erroneously written as “doi: 10.1038/s41598-022-13710-2”. It should be “doi: 10.1038/s41598-022-14512-3”.
The DOI for Reference 38 was erroneously written as “doi: 10.1038/s41598-022-14512-3”. It should be “doi: 10.3389/fpsyt.2021.687546”.

Reference 35 was a duplicate of Reference 14. All citations for Reference 35 have been updated to point to Reference 14, and all subsequent citations have been renumbered.

The original version of this article has been updated.

